# Primary Genital HSV-2 Infection in a Resource-Limited Setting: Diagnostic Challenges and Clinical Implications

**DOI:** 10.7759/cureus.95820

**Published:** 2025-10-31

**Authors:** Rachma O Maksud, Nurul Rahma Putri P Antuke, Taufiq Qurrohman

**Affiliations:** 1 Department of Dermatology and Venerology, Toto Kabila General Hospital, Gorontalo, IDN; 2 Department of Dermatology and Venerology, Faculty of Medicine, Universitas Negeri Gorontalo, Gorontalo, IDN; 3 Department of Dermatology, Faculty of Medicine, Universitas Negeri Gorontalo, Gorontalo, IDN

**Keywords:** antiviral therapy, clinical diagnosis, genital herpes, herpes simplex virus 2, recurrent infection, resource-limited settings, sexually transmitted infection

## Abstract

Herpes simplex virus type 2 (HSV-2) is a chronic sexually transmitted infection and the leading cause of genital ulcer disease worldwide. The virus establishes lifelong latency with periodic reactivation, resulting in recurrent lesions and a substantial clinical and psychosocial burden. Moreover, HSV-2 increases susceptibility to HIV infection, underscoring its global public health relevance. We report a 27-year-old male who presented to the dermatovenereology clinic with a three-day history of painful penile ulcers following recent unprotected sexual contact. Physical examination revealed multiple irregular, lenticular ulcers with erythematous bases and crusting on the corpus penis and corona glandis. The patient was alert, afebrile, and without systemic symptoms. Due to limited diagnostic resources, confirmatory testing by polymerase chain reaction (PCR) or serology was unavailable. A clinical diagnosis of primary genital HSV-2 infection was made after excluding syphilis and lymphogranuloma venereum (LGV). Treatment with oral acyclovir, cefadroxil, and mefenamic acid, supplemented by topical gentamicin and counselling on recurrence risk and safe sexual practices, led to complete lesion resolution within 14 days and no recurrence at six-week follow-up. This case highlights the dual challenge of managing HSV-2 in resource-limited settings: the lifelong, recurrent nature of the infection and the absence of confirmatory diagnostic tools. These limitations hinder accurate case identification and contribute to the underrecognition of HSV-2 prevalence. Early clinical recognition, prompt antiviral initiation, and strengthened patient education remain essential for mitigating morbidity, preventing recurrence, and reducing transmission in low-resource environments.

## Introduction

Herpes simplex virus type 2 (HSV-2) remains a significant public health issue globally, primarily due to its high prevalence and the recurrent nature of associated infections [1]. The virus poses substantial health risks, particularly as it can exacerbate the vulnerability of individuals to HIV and other sexually transmitted infections (STIs). The epidemiology of HSV-2 highlights the need for comprehensive strategies in diagnosing, treating, and preventing its transmission, particularly in populations at higher risk, such as pregnant women, individuals with immunocompromised conditions, and men who have sex with men (MSM) [2].

Studies indicate that HSV-2 prevalence is notably high among populations at risk, including MSM and individuals living with HIV. For instance, a study among HIV-positive MSM, HSV-2 seroprevalence stands at 55%, while 17% is observed among HIV-negative peers, suggesting a correlation between HSV-2 and HIV infection [1]. Furthermore, in a cohort of pregnant women in Rio de Janeiro, 59.7% were found to be seropositive for HSV-2 [2].

The co-infection of HSV-2 with HIV significantly contributes to the transmission dynamics of both viruses. The study on patients with genital ulcer disease in Malawi found that HSV-2 ulcers were present in 72% of cases, highlighting the association with HIV prevalence, particularly in recurrent instances of HSV-2 [3]. Public health strategies addressing HSV-2 could therefore enhance the management of HIV transmission [1]. Recurrence of HSV-2 infections not only causes physical discomfort but also increases the risk of psychological distress and social stigma associated with herpes infections. Pregnant women infected with HIV have an increased risk of severe and recurrent HSV-2 infections, with significant implications for prenatal health and neonatal outcomes [2]. In addition, challenges exist in managing acyclovir-resistant HSV infections, particularly in immunocompromised patients such as those undergoing stem cell transplants, complicating treatment options and necessitating alternative approaches [4,5].

The literature emphasizes the importance of routine screening for HSV infections, especially in women presenting with symptoms of vaginitis. In a study involving women seeking care for vaginitis, up to 30% were found to be seropositive for HSV-2, indicating a need for better diagnostic practices [6]. Public health campaigns should focus on increasing awareness and education regarding safe sexual practices to reduce HSV transmission rates [3]. Despite the challenges posed by HSV-2, research into effective vaccines is underway. Studies indicate promising results using adjuvanted HSV-2 subunit vaccines [6]. Future strategies focusing on inducing robust mucosal immunity in the genital tract hold potential for significant advancements in preventing HSV-2 infections [7].

This case report presents a 27-year-old male with recurrent HSV-2 infection manifesting as multiple painful genital ulcers. It underscores the diagnostic challenges faced in resource-limited healthcare settings where confirmatory tests are unavailable, leading to reliance on clinical judgment alone. By situating this case within the broader epidemiological and clinical context, this report aims to emphasize the importance of strengthening diagnostic infrastructure, enhancing physician awareness, and recognizing HSV-2 not only as an infectious disease but also as a public health burden in lower-middle-income countries (LMICs).

## Case presentation

A 27-year-old unmarried male presented to the Dermatology and Venereology outpatient clinic with a chief complaint of painful sores on his penis for the past three days. The patient reported that the lesions began as small, painful spots that rapidly evolved into multiple ulcerations associated with constant pain, particularly exacerbated by water exposure during bathing. He denied dysuria, fever, or malaise and had no prior history of similar episodes, suggesting that this was his first clinically recognized episode. Notably, the patient admitted to recent unprotected sexual contact approximately three days before symptom onset, which he believed may have triggered the current illness. He had attempted self-medication with a topical ointment without improvement. His past medical history was unremarkable, with no known chronic diseases, immunosuppressive conditions, or previous STIs. He denied alcohol use, smoking, or recreational drug use and reported no known drug or food allergies.

**Figure 1:**
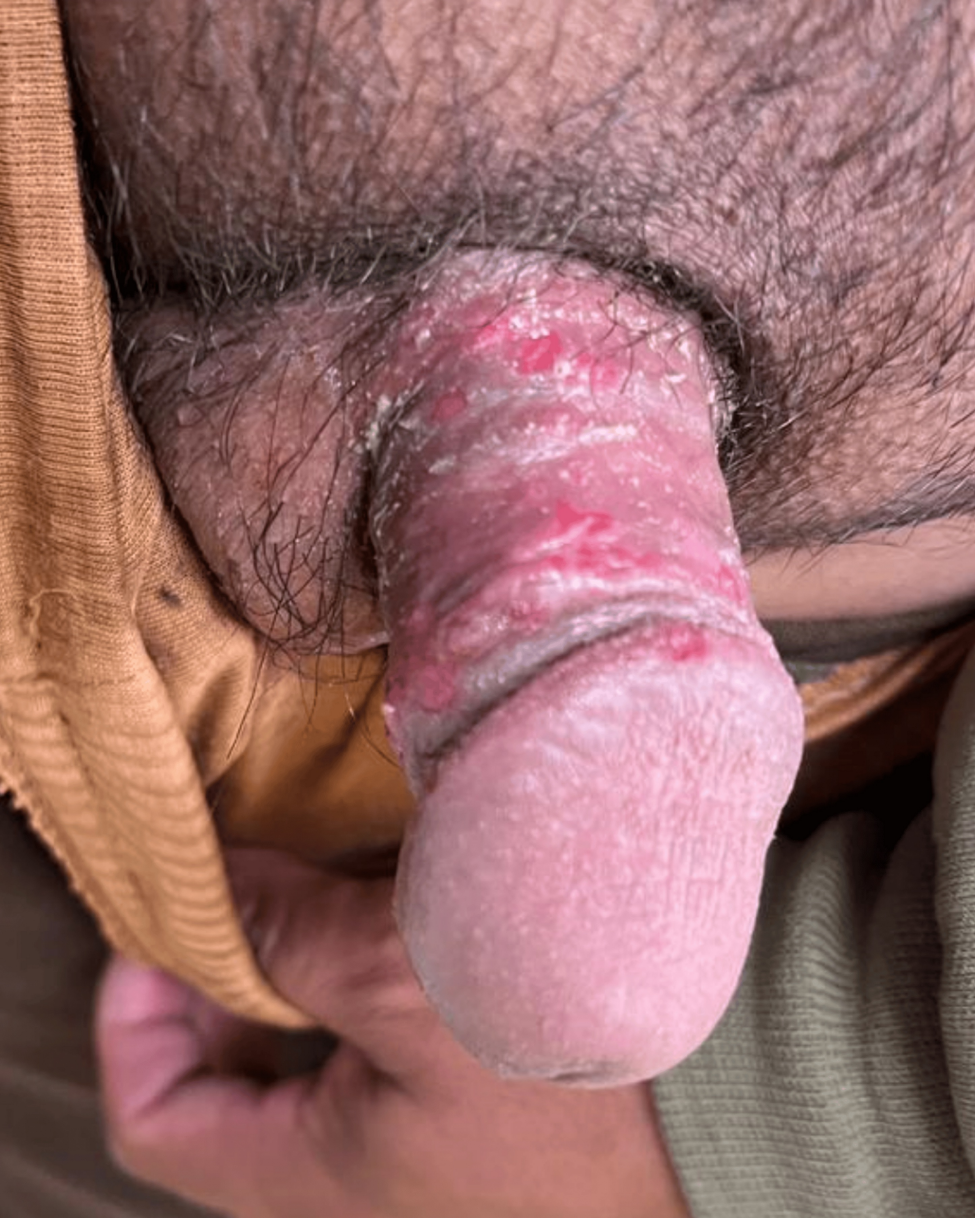
Multiple painful ulcerative lesions of genital herpes simplex virus type 2 (HSV-2) on the penis. Clinical photograph showing irregularly shaped, shallow ulcerations with erythematous bases and overlying crusts localized on the proximal shaft and corona of the penis. The ulcers appear grouped, with surrounding erythema and mild exudation, consistent with a primary episode of HSV-2 infection.

On physical examination, the patient was alert, afebrile (temperature 36.8°C), not pale or jaundiced, but appeared moderately ill due to pain. His pulse rate was 86 beats per minute, blood pressure 118/76 mmHg, and respiratory rate 18 breaths per minute. Dermatological examination revealed multiple lenticular ulcers located on the corpus penis and corona glandis penis, as shown in Figure 1. The ulcers were irregular but tended towards round shapes, with circumscribed borders, clean erythematous bases, and partial crust formation. The lesions were tender to palpation, and no induration or necrosis was observed. No extragenital lesions or inguinal lymphadenopathy were present. Based on the characteristic clinical presentation of painful grouped ulcers localized to the genitalia following recent unprotected sexual exposure, a diagnosis of genital HSV-2 infection was made. Differential diagnoses such as syphilis, which typically presents with a painless chancre, and lymphogranuloma venereum (LGV), which generally begins with unnoticed or painless lesions, were considered less likely in this case.

Due to the limitations of the healthcare setting, no comprehensive laboratory investigations could be performed. Advanced confirmatory diagnostics such as polymerase chain reaction (PCR), viral culture, or serological assays (IgM/IgG anti-HSV) were unavailable at the facility. However, the patient was screened for HIV using a rapid test, which yielded a negative result. Consequently, the diagnosis relied primarily on clinical features, underscoring the challenges of managing STIs in resource-constrained environments.

The patient was treated with oral acyclovir 400 mg three times daily for five days, combined with oral mefenamic acid 500 mg for analgesia over three days, and oral cefadroxil 500 mg for five days to prevent secondary bacterial infection. In addition, gentamicin ointment was prescribed twice daily for one week. Supportive and non-pharmacological management included counseling regarding the natural course of the disease, the risk of recurrence, and the importance of sexual abstinence until complete resolution of lesions. The patient was also advised to disclose his infection status to his sexual partner and to use condoms consistently in future sexual activity to reduce the risk of transmission, with an emphasis that condom use lowers but does not eliminate the risk due to uncovered areas that may shed the virus.

The prognosis for this patient was considered good regarding life and function, as the genital ulcers showed marked improvement within seven days and complete healing by approximately 14 days after initiating antiviral therapy. The patient was followed up for six weeks, during which no recurrence was observed. However, the long-term prognosis regarding cure remains uncertain, given the lifelong persistence of HSV in sensory ganglia and the potential for future recurrence. This case underscores the significant clinical burden of HSV-2 infection in young adults and highlights the diagnostic and management challenges faced in limited-resource healthcare settings where confirmatory laboratory testing is unavailable.

## Discussion

Genital HSV-2 infection remains a global public health concern due to its chronic, recurrent, and psychologically distressing nature. Beyond being a common cause of genital ulcer disease, HSV-2 contributes to significant morbidity through recurrent lesions, chronic pain, and neuroinflammatory sequelae that influence both physical and emotional well-being. The virus establishes lifelong latency in sensory ganglia, with periodic reactivation driven by immune, hormonal, or psychological stressors. Emerging evidence implicates HSV-2 in neuroinflammatory pathways mediated by microRNA regulation of immune responses, which may explain the persistence and recurrence patterns observed clinically [8]. Thus, management extends beyond symptom control to encompass the infection’s psychosocial and neuroimmunological dimensions.

In the present case, a 27-year-old unmarried male presented with a primary episode of genital HSV-2 infection following unprotected sexual contact. Lesions and multiple, painful, erythematous ulcers on the penis were typical of a first episode, characterized by localized tenderness without systemic manifestations. The absence of dysuria or generalized symptoms is consistent with mild to moderate presentations seen in immunocompetent adults. Importantly, the negative HIV rapid test ruled out immunosuppression as a predisposing factor. The patient achieved complete healing within two weeks of initiating oral acyclovir, with no recurrence at the six-week follow-up, underscoring the effectiveness of timely antiviral therapy in the first episode of HSV-2.

However, the diagnosis in this case relied exclusively on clinical evaluation due to limited access to confirmatory tests such as PCR, viral culture, or serology. This reflects a persistent challenge in resource-limited settings, where clinicians must depend on clinical acumen to distinguish HSV-2 from other ulcerative STIs such as syphilis or LGV. The absence of diagnostic capacity not only hampers accurate case identification but also limits opportunities for counseling, surveillance, and partner notification critical components in STI control. These constraints perpetuate cycles of delayed care and ongoing transmission, highlighting the urgent need for scalable, low-cost diagnostic solutions.

The psychological impact of HSV-2 infection is often underestimated yet profound. The diagnosis can precipitate distress, guilt, and fear of stigma, especially in young adults navigating sexual and social relationships. The recurrent and visible nature of genital lesions compounds anxiety and can lead to social withdrawal or diminished self-esteem. Studies have consistently shown that HSV-2 adversely affects multiple dimensions of health-related quality of life (HRQOL), including emotional stability, intimacy, and overall well-being [9-11]. The infection’s chronicity transforms what might appear to be an acute dermatological problem into a long-term psychosocial condition. Moreover, economic and productivity losses add a further burden, particularly in LMICs, where out-of-pocket treatment costs are substantial [12].

Gender differences also shape the psychosocial experience of HSV-2. Women, in particular, face greater social stigma and lower sexual quality of life scores than men, possibly due to gender norms and unequal power dynamics in relationships [13]. Addressing such disparities requires public health strategies that extend beyond clinical management to encompass community education, destigmatization, and equitable access to care.

From a therapeutic perspective, suppressive antiviral therapy with agents such as valacyclovir remains central to reducing recurrence frequency and viral shedding, thereby improving QOL and lowering transmission risk. Early initiation of therapy, as in this case, can limit lesion duration and symptom severity while preventing secondary infections [14]. However, in many low-resource environments, access to long-term suppressive therapy is restricted by cost and drug availability, leaving patients dependent on episodic treatment that addresses acute symptoms but not recurrence prevention. This gap in care underscores the intersection of socioeconomic and clinical determinants in HSV-2 outcomes.

Recent diagnostic innovations offer a path forward. The Smart Cup platform, which integrates smartphone-based detection with a phase-change material for temperature regulation, enables nucleic acid amplification without advanced laboratory equipment [15]. Similarly, a polypropylene pouch colorimetric assay allows naked-eye visualization of HSV-1 and HSV-2 DNA using hydroxy naphthol blue dye, eliminating the need for complex extraction steps [16]. Such portable, affordable technologies could transform diagnosis in rural and field settings, facilitating earlier treatment and more accurate epidemiologic surveillance. Nevertheless, issues of test reliability, reagent stability, and user training must be addressed before widespread implementation.

Preventive strategies remain an indispensable component of HSV-2 control. Behavioral interventions promoting consistent condom use continue to demonstrate variable success in reducing STI transmission [13,16]. While condoms significantly reduce but do not eliminate the risk of HSV-2 transmission, barriers such as accessibility, misinformation, and social stigma persist. Integrating diagnostic and therapeutic advances with behavioral education tailored to cultural contexts can yield more sustainable prevention outcomes. Furthermore, counseling patients on the chronic nature of HSV-2, partner disclosure, and safe sexual practices is essential to minimize transmission and improve psychological adaptation.

This case also reflects a broader epidemiological reality, as HSV-2 remains one of the most prevalent STIs globally. Recent modelling analyses have estimated that in 2020 alone, approximately 25.6 million new HSV-2 infections occurred among individuals aged 15-49 years, contributing to a global prevalence exceeding 519 million, or 13.3% of this age group. The burden is disproportionately higher among women and individuals in LMICs, where diagnostic and preventive services remain limited [17]. These findings underscore that the presented case of an initial symptomatic episode in a young, otherwise healthy male represents a small but important component of a massive, under-recognized global disease burden.

Clinically, the presentation in this case highlights the challenges of managing HSV-2 in resource-limited settings. The absence of confirmatory laboratory testing forced clinicians to rely on classical clinical diagnosis, which, while practical, risks both under- and over-diagnosis due to the overlap of genital ulcer disease etiologies. Globally, such diagnostic constraints contribute to a significant underestimation of HSV-2 incidence, particularly in LMICs where confirmatory tests such as PCR are either unavailable or unaffordable. This reinforces the urgency for wider implementation of low-cost, point-of-care molecular diagnostics that are adaptable to decentralized health systems.

From a public health standpoint, the case further illustrates the intersection between clinical outcomes and psychosocial determinants. For first-time HSV-2 patients, acute distress and stigma may amplify psychological morbidity. In patriarchal societies or culturally conservative regions, men may underreport symptoms or delay seeking care due to embarrassment or fear of social repercussions. Early counseling, therefore, becomes essential not only to address sexual behavior and transmission risks but also to reduce self-stigma and improve adherence to preventive measures.

Importantly, epidemiological data demonstrate that HSV-2 plays a synergistic role in amplifying HIV transmission risk, particularly in populations with high sexual network connectivity or limited condom access. Given that HSV-2 increases the susceptibility to HIV acquisition threefold, the failure to control HSV-2 spread indirectly undermines broader STI and HIV prevention strategies. For this reason, the WHO has emphasized the need for integrated surveillance and the development of prophylactic and therapeutic vaccines against HSV-2 [18].

In the context of this patient, the prompt initiation of acyclovir contributed to rapid clinical recovery, reflecting the importance of early antiviral therapy in mitigating lesion duration and transmission potential. However, the lifelong persistence of HSV within sensory ganglia means that recurrence remains a possibility. As shown in global models, recurrent genital ulcer disease (GUD) episodes affect nearly 188 million people annually, a figure that underscores both the chronic nature of infection and the cumulative psychosocial and economic toll on affected individuals [17].

Ultimately, this case reinforces the dual challenge of HSV-2 management: addressing the immediate clinical needs of individual patients while recognizing the immense public health implications of an infection that remains largely invisible yet widespread. Strengthening diagnostic capacity, expanding antiviral access, and promoting behavioral interventions tailored to cultural contexts are essential steps toward reducing HSV-2 morbidity in low-resource settings. The ongoing global estimates highlight an urgent need for integrated STI control programs and renewed investment in HSV vaccine research as strategic measures to curb transmission, improve sexual health outcomes, and alleviate the psychosocial burden associated with this lifelong infection.

## Conclusions

This case highlights the clinical and psychosocial impact of a first episode of genital HSV-2 infection in a young adult. The painful ulcers caused notable discomfort and distress, reflecting the significant burden even a single episode can impose. Diagnostic challenges in resource-limited settings, where confirmatory tests such as PCR or serology are unavailable, force clinicians to rely solely on clinical judgment. Limited access to antiviral therapy further complicates management and increases the risk of recurrence. Strengthening diagnostic capacity, ensuring affordable antiviral access, and improving clinical awareness are essential to reduce the personal and public health impact of HSV-2 in low-resource environments.
